# Extraversion, Neuroticism, and Employee Voice: A Conservation of Resources Perspective

**DOI:** 10.3389/fpsyg.2020.01281

**Published:** 2020-06-26

**Authors:** Jia Li, Sen Xu

**Affiliations:** ^1^Business School, Nanjing University, Nanjing, China; ^2^School of Economics and Management, Nanjing Tech University, Nanjing, China

**Keywords:** employee voice, extraversion, neuroticism, emotional exhaustion, conservation of resources theory

## Abstract

Drawing on the Conservation of Resources (COR) theory, we constructed a model about how two of Big-Five personality traits, extraversion and neuroticism, respectively, influence employee voice through an indirect effect of emotional exhaustion. We distributed two wave surveys to 435 employees and their supervisors in a Chinese state-owned bank. Our analyses indicated that extraversion had a positive indirect effect on employee voice *via* emotional exhaustion, whereas neuroticism had a negative indirect effect on employee voice *via* emotional exhaustion. We concluded by discussing our theoretical implications with research on employee voice and COR. Additionally, we discussed our practical implications for managerial practices and for the general public.

## Introduction

“Who speaks up at work?” is a fundamental question in research on employee voice, which refers to a “promotive behavior that emphasizes expression of constructive challenge intended to improve rather than merely criticize” (Van Dyne and LePine, 1998, p. 109). Research has taken a disposition perspective to examine whether voice emerges among employees with certain individual dispositions (e.g., LePine and Van Dyne, 2001; Avery, 2003; Nikolaou et al., 2008). As the Big-Five personality traits model is one of the most widespread personality frameworks (Costa and McCrae, 1992), researchers have examined how this model influences employee voice (Morrison, 2011, 2014; Klass et al., 2012; Chamberlin et al., 2017; Zare and Flinchbaugh, 2019).

While current studies have somewhat answered who are likely (not) to voice, better understanding remains in needs to explain a more important “why” question, which aims to explore the mechanisms through which employees with specific Big-Five personality traits are likely (not) to voice at work. As such, we utilized the Conservation of Resources (COR) theory (Hobfoll, 1989; Halbesleben et al., 2014; Hobfoll et al., 2018) to examine the relationship between two Big-Five personality traits (extraversion and neuroticism) and employee voice through an indirect effect of emotional exhaustion. Empirically, while extraversion is the most positive predictor of employee voice in the Big-Five personality model, neuroticism is the least relevant predictor and has no significant effect on voice (Chamberlin et al., 2017; Zare and Flinchbaugh, 2019). Accordingly, our investigation on these two traits could coincidentally exhibit whether the non-relationship between neuroticism and voice is because of omitted mediating mechanisms. Theoretically, although scholars have proactively responded to calls for studies on how emotion processes affect employee voice (Morrison, 2014), existing research has concentrated on how the emotional mechanism facilitates rather than hinders voice (Chamberlin et al., 2017). Specifically, the role of negative emotional processes such as emotional exhaustion (Qin et al., 2014) has been far away from achieving a consensus. In fact, emotional exhaustion serves as a reaction to excessive stimuli to deplete the energy and resource of emotion in the employees’ daily work life (Maslach, 1993). Compared to other Big-Five traits, extraversion and neuroticism are personality stimuli to positive emotions and negative emotions, respectively (Larsen and Ketelaar, 1991). Individual differences in extraversion and neuroticism could influence the degree of employees’ emotional exhaustion. Emotionally exhausted employees may cope with such a loss of emotional resources by withholding their voice at work. To examine this mediating model, we designed a two-wave survey to 435 employees and their supervisors working in a Chinese state-owned bank.

Our study provides certain theoretical contributions and practical implications. First, we investigated a largely ignored mediating psychological mechanism behind the relationship between individual dispositions and employee voice. This omission deserves considerable attention because researchers have urged a broader view of why certain employees engaged (not) in voice (Van Dyne et al., 2003; Morrison, 2014). Exploring the underlying intervening mechanisms could integrate and move forward the existing studies on the disposition–voice relationship. Second, we enriched current understanding of the nature, mechanism, and value of emotional resources on employee voice. Regulating their own emotions has been recognized as one key psychological process about why employees speak up at work (Van Dyne et al., 2003; Morrison, 2014). We underscore the importance of emotional regulations in employee voice by readdressing employee voice as a coping mechanism of resource conservation (Ng and Feldman, 2012). Third, our study provided certain practical implications for prosocial voice. We specified prescriptions for business organizations to build an open culture, prescriptions for managers to encourage employee voice at work, and prescriptions for what employees can self-enhance their voice. We also advised how civil servants are motivated by policy makers, public organizations, and themselves to serve the general public.

## Theoretical Backgrounds and Hypotheses

### Big-Five Personality Traits and Employee Voice

Research on employee voice has been burgeoning. Given that employee voice could inspire team learning, increase unit effectiveness, decrease turnover, and promote organizational change and innovation (for reviews, see Morrison, 2011, 2014; Klass et al., 2012), prior research has continuously explored the antecedents and reasons why employees voice at work (Morrison, 2011, 2014). In doing so, some studies have looked at how employee voice is influenced by individual dispositions, which refer to “fundamental capacities and characteristics of individuals that influence how they tend to feel, think, and ultimately behave” (Chamberlin et al., 2017, p. 7–8).

The Big-Five personality traits model has received most attention among different types of individual dispositions. Prior studies have mainly examined the direct relationship between the Big-Five personality traits and employee voice. Results indicate this relationship is complicated. For example, LePine and Van Dyne (2001) designed a laboratory study to examine the extent to which the Big-Five personality traits influence voice among 276 junior and senior students in the United States. Results show that extraversion and conscientiousness relate positively to voice, whereas neuroticism and agreeable negatively relate to voice. Nikolaou et al. (2008) distributed surveys to 334 professionals who were enrolled in graduate management courses in Greece. They found that conscientiousness associates positively with employee voice toward their supervisors, whereas neuroticism associates negatively with employee voice toward their supervisors. But their findings did not support a significant extraversion–voice relationship. Crant et al. (2011) invited a total of 244 MBAs and undergraduates to assess their own Big-Five personality traits and recorded their voice in the classroom. They also found that extraversion and conscientiousness connect positively with expression of voice, whereas the neuroticism–voice relationship does not exist. Recent research has detailed the Big-Five–voice relationship with regard to multiple facets of employee voice. Liu et al. (2014) sent surveys to 203 employee-supervisor dyads in a Chinese information technology corporation. They found that both extraversion and conscientiousness positively predict prohibitive voice and that neuroticism negatively predicts prohibitive voice. Maynes and Podsakoff (2014) substantially extended our understanding of the Big-Five–employee voice relationship in a sample of executive MBAs in the United States. A complex relationship emerged between each of the Big-Five personality traits and four different types of voice (supportive, constructive, defensive, and destructive). Chamberlin et al. (2017) conducted a meta-analysis on the Big-Five–employee voice relationship. Their findings suggested that extraversion is the most relevant Big-Five personality trait to employee voice and that neuroticism is the least relevant Big-Five personality trait to employee voice. Recently, Zare and Flinchbaugh (2019) also used the meta-analysis method to consistently find that extraversion is a good indicator of voice, whereas neuroticism is not.

Despite ample empirical evidence, much effort needs to be done before we conclude the Big-Five–employee voice relationship. There is a “largely missing” on the “coherent theoretical framework” for “conceptual synthesis and integration” about why individual dispositions influence employee voice (Morrison, 2014, p. 393). Scholars may bring new perspectives and explore its mediating mechanisms. More explicitly, researchers may respond actively to the why question as under what intervening mechanisms are employees with specific individual dispositions likely (or not) to voice at work.

### Extraversion, Neuroticism, and Employee Voice From a COR Theory

We rethink about the Big-Five–employee voice relationship from a view of COR theory. According to the COR theory, employees conserve and acquire personal (e.g., time, physical energy, emotional energy) and external resources (e.g., coworker support, leadership style, organizational culture) to prevent and compensate for possible or actual loss of these resources in stressful situations (Hobfoll, 1989; Halbesleben et al., 2014; Hobfoll et al., 2018). As such, the COR theory has been recognized as a fundamental theory to explain how employees are motivated to deal with stress under challenging work circumstances and requirements such as employee voice (Hobfoll et al., 2018). Specifically, employees will engage in voice behaviors to avoid resource losses as loss could result in a negative influence on their well-being (Ng and Feldman, 2012). In line with the COR theory, prior studies have examined how employees use voice as a means of resource protection and acquisition in response to workplace stressors (Ng and Feldman, 2012; Carnevale et al., 2018; Zhou et al., 2019). Personal characteristics are traditionally seen as personal resources that employees could bring to their daily work lives for assisting stress resistance (Hobfoll, 1989). Among a wide range of personal characteristics, personality traits represent relatively stable aspects of employees and determine how they respond to ambivalent stimuli and the types of coping strategies through which employees implement (Halbesleben et al., 2014). Scholars have identified that personality traits could explain almost one-fifth of the variance in voice (Chamberlin et al., 2017). Although the Big-Five personality traits are the most representative and valid personality trait model, their influence on employee voice varies significantly (Zare and Flinchbaugh, 2019). This variation is due to a lack of deep exploration to theoretically unfold the mechanism of “why” the Big-Five personality traits affect employee voice. In an attempt to move this stream of research forward, our investigation used the COR theory to frame voice as a coping strategy that helps employees resolve the depletion of personal sources, which are indicated by Big-Five personality traits.

Our study centered on two of Big-Five personality traits: extraversion and neuroticism, which coincidentally represent stimuli of employees’ positive emotions and negative emotions, respectively. In addition, we used emotional exhaustion as a criterion of psychological strain to indicate the extent to which these employees deplete their emotional resources and that ultimately influence employee voice as a coping strategy to their depletion of emotional resources.

#### Extraversion and Emotional Exhaustion

Extraversion describes the extent to which individuals are active, assertive, energetic, enthusiastic, outgoing, and talkative (Costa and McCrae, 1992). Extravert employees may stay with people to seek and enjoy excitement, whereas introvert employees prefer to stay alone with reservation, quietness, and independence. Extraversion represents an individual characteristic to perceive and respond to position emotional stimuli, thereby causing long-term positive emotions (Larsen and Ketelaar, 1991; Rusting, 1998; Judge and Zapata, 2015).

Drawing from the COR theory (Hobfoll, 1989; Halbesleben et al., 2014; Hobfoll et al., 2018), extraversion relates negatively to emotional exhaustion. Emotional exhaustion is the core feature of job burnout (Maslach et al., 2001) and refers to “feelings of being emotionally overextended and depleted of one’s emotional resources” (Maslach, 1993, p. 20–21). Sources of emotional exhaustion at work could be workload, time pressure, lack of job control, and work–family conflict (Maslach et al., 2001).

By viewing extraversion as personal stimuli of positive emotions, we connect extraversion with the state of emotional resource depletion, which is illustrated by emotional exhaustion. The COR theory suggests that, when individuals experience insufficient emotional resources, they may perceive emotional exhaustion (Wright and Cropanzano, 1998). Because extravert employees are outgoing and participate in groups, they may maintain their high levels of positive emotions at work despite certain job stressors. As such, these extravert employees may experience low levels of emotional exhaustion. By contrast, introvert employees keep quiet and alone in teams. They may not exhibit high levels of positive emotions even if they experience certain joy and achievement. Based on this psychological condition, introverts may feel high levels of emotional exhaustion.

#### Neuroticism and Emotional Exhaustion

Neuroticism shows individual differences on emotional adjustment (Costa and McCrae, 1992). While neurotic persons, or individuals with low emotional stability, are anxious, self-pitying, tense, touchy, unstable, and worrying, emotional stables are confident, calm, and relax. Research has viewed neuroticism as one trait about the extent to which individuals feel negative emotional stimuli, therefore leading to long-term negative emotions (Larsen and Ketelaar, 1991; Rusting, 1998; Judge and Zapata, 2015).

Since the COR literature has examined neuroticism as an employee’ stimuli of negative emotions (Halbesleben et al., 2014; Beehr et al., 2015), we suggest a positive relationship between neurotic employees and their levels of emotional exhaustion. As these neurotic employees are emotionally sensitive to job stressors, they could experience negative emotions (e.g., anxiety, anger, fear) more frequently and intensively than their emotionally stable colleagues (Costa and McCrae, 1992). These neurotic employees may not effectively use coping strategies to release their negative emotions (Hampson, 2012; Bowling and Jex, 2013) and accordingly increase the possibility to deplete their emotional resources.

#### The Indirect Effect of Emotional Exhaustion

Emotional exhaustion could significantly prevent employees from reaching their job demands and fulfilling performance standards (Maslach et al., 2001). Similarly, we consider emotional exhaustion as a state of being overworked and resource depletion and employee voice as a coping behavior that resolves resource losses. Regarding employee voice, the COR literature has explained how personal resource loss, which is indicated by emotional exhaustion, could influence employee voice *via* two tenets (Ng and Feldman, 2012; Qin et al., 2014). While the resource conservation tenet suggests that these emotional exhausted employees may not speak up in order to impede further emotional resource losses, the resource acquisition tenet hints that these emotional exhausted employees are likely to speak up in order to obtain extra emotional resources to alleviate or compensate for emotional resource losses (Hobfoll, 1989; Ng and Feldman, 2012).

Our study takes the resource conservation tenet to suggest that emotional exhausted employees are likely to voice in terms of three reasons. First, the principle of resource conservation is more significant than that of resource acquisition. A lack of resources results in defensive responses to protect their remaining resources (Halbesleben et al., 2014; Hobfoll et al., 2018). Second, recent meta-analysis supports the resource conservation strategy in explaining employee voice. When the employees suffered from work stressors, their feelings of resource depletion encourage them to provide constructive suggestions because of avoiding further resource losses (Ng and Feldman, 2012). Third, the resource acquisition tenet could be implemented to motivate emotional exhausted employees to speak up under certain work and team boundary conditions (Qin et al., 2014).

Furthermore, our early justification explained that whereas extraversion relates negatively to emotional exhaustion, neuroticism relates positively to emotional exhaustion. In addition to our previous argument on the negative relationship between emotional exhaustion and employee voice, we posit that

H1. Extraversion has an indirect effect on employee voice through emotional exhaustion. Extraversion negatively associates with emotional exhaustion, which in turn negatively associates with employee voice.
H2. Neuroticism has an indirect effect on employee voice through emotional exhaustion. Neuroticism positively associates with emotional exhaustion, which in turn negatively associates with employee voice.

## Materials and Methods

### Participants and Procedures

We collected data in a state-owned bank located in a provincial capital of China. The bank had nearly 80 branches with almost 1400 employees. Human resource department helped send our recruitment information to these branches. Managers from 60 branches agreed to distribute our questionnaires. In order to minimize the common method bias, we conducted surveys to two sources at two different times on site. Identification numbers were used to match branch employees and their voice behavior scores rating from the branch managers. All participants were asked to fill the survey voluntarily. We informed the participants that their identities would be kept anonymously and confidentially.

At Time 1, we sent the first survey to 670 employees in these 60 branches, asking the employees to provide information about their demographic characteristics, personal characteristics, and work characteristics. Three weeks later, because one branch manager did not want to participate, we dropped our data collection in his branch. We distributed our second questionnaire to the other 59 branch managers and 493 employees who responded in the first round. We asked the employees to evaluate their emotional exhaustion. Meanwhile, their supervisors rated each employee’s voice behavior. We received responses from 480 employees and their 59 supervisors.

After eliminating incomplete and unmatched questionnaires, our final sample consisted of 435 employees and 58 supervisors (an average of 7.5 employees per supervisor), with a response rate of 64.9% for employees and 96.7% for supervisors, respectively. Of these 435 employees, the mean age and working tenure were 29 and 6.79 years, respectively. Two-thirds of the employees were female (66.4%), and four-fifths were well educated (81.1% had completed undergraduate degrees or above).

### Measures

We adopted existing scales to measure all primary variables in Mandarin. Two independent bilingual researchers with majors in management conducted translations and back-translations (Brislin, 1986). Then, we invited three bank staff to conduct pilot studies before distributing our survey.

#### Extraversion

We used Mini-Markers developed by Saucier (1994) to measure extraversion. At Time 1, the participants rated how accurately these words described themselves in eight adjectives, ranging from one (extremely inaccurate) to seven (extremely accurate). Sample items were “bold,” “talkative,” and “shy.” Cronbach α for this scale was 0.72.

#### Neuroticism

We also used eight items adopted from Saucier’s (1994) Mini-Markers to measure neuroticism. At Time 1, we asked the employees to rate how accurately the following adjectives describe themselves, ranging from one (extremely inaccurate) to seven (extremely accurate). Sample items were “envious,” “moody,” and “relaxed.” Cronbach α for this scale was 0.79.

#### Emotional Exhaustion

Six items for emotional exhaustion were adopted from Wharton’s (1993) scale. At Time 2, the employees rated their feelings about emotional exhaustion in the workplace, ranging from one (strongly disagree) to seven (strongly agree). Sample items were “I feel emotionally drained from my work” and “I feel used up at the end of the workday.” Cronbach α for this scale was 0.95.

#### Employee Voice

We examined employee voice using Van Dyne and LePine’s (1998) six-item scale. At Time 2, the supervisors rated the individual voice behavior of each employee, ranging from one (strongly disagree) to seven (strongly agree). A sample item was “This employee develops and makes recommendations concerning issues that affect this work group.” Cronbach α for this scale was 0.94.

#### Control Variables

We controlled for four demographic variables suggested by prior studies (Kakkar et al., 2016; Venkataramani et al., 2016): (1) age (years); (2) gender (0 = male, 1 = female); (3) education level (1 = high school/college degree, 2 = undergraduate degree, 3 = postgraduate degree); and (4) organizational tenure (years).

### Analytic Strategy

Although our proposed model operated at the individual level, our data structure was nested because one manager rated multiple employees on their voice behaviors (average number of employees per manager = 7.5). Hence, our observations potentially violated the independence assumption. We checked for the presence of nesting effects (Bliese, 2000). One-way analysis of variance result indicated systematic differences in supervisors’ ratings of employees’ voice behaviors (*p* < 0.01; intraclass correlation = 0.44), reflecting that there is 44% residual variance between supervisors. Accordingly, multilevel structural equation modeling (MSEM) could examine the effects of the individual-level variables while accounting for the non-interdependence of observations within groups. We used MSEM *via* Mplus 7 (Muthén and Muthén, 1998-2015) to test the overall model. Furthermore, the indirect effects described in the hypotheses require calculation of non-normally distributed compound coefficients. Because bootstrapping is not available in MSEM *via* Mplus (Muthén and Muthén, 1998-2015), we used Monte Carlo simulation with 20,000 resamples to calculate the 95% confidence intervals (CIs) in the program R for the mediation hypotheses (Selig and Preacher, 2008).

## Results

### Preliminary Analyses

We conducted confirmatory factor analyses to assess the distinctiveness of four constructs (extraversion, neuroticism, emotional exhaustion, and voice behavior). To enhance the reliability and parsimony of our model, item parcels were created for extraversion (eight items) and neuroticism (eight items). Each factor was defined by four parcels, with each parcel being created by sequentially summing items assigned based on the highest to lowest item-total corrected correlations, so as to obtain less free parameters to estimate and to reduce the sources of sampling error (Coffman and MacCallum, 2005). The model yielded good fit to the data (χ^2^ (163) = 457.11, *p* < 0.01, Root Mean Square Error of Approximation (RMSEA) = 0.06, Comparative-Fit Index (CFI) = 0.95, Tucker-Lewis Index (TLI) = 0.94, Standardized Root Mean Square Residual (SRMR) = 0.05). All the indicators loaded from the latent constructs were significant, confirming their convergent validity. Then, we contrasted the four-factor model against three alternative models. Table 1 presented the models’ fit indices. These model comparison results showed all the alternative models yielded poorer fits to the data and significantly worse than the four-factor model. Thus, we concluded that the theoretical constructs had discriminant validity.

**TABLE 1:** Comparison of measurement models.

	χ ^2^	*df*	RMSEA	CFI	TLI	SRMR	△χ ^2^ (△*df*)
Four-factor model^a^	457.11	163	0.06	0.95	0.94	0.05	−
Three-factor model^b^	733.04	166	0.09	0.91	0.89	0.08	275.93**(3)
Two-factor model^c^	1322.17	168	0.13	0.81	0.78	0.11	865.06**(5)
One-factor model^d^	3481.24	169	0.21	0.44	0.37	0.21	3024.13**(6)

*N* = 435. *p < 0.05. **p < 0.01 (two-tailed). ^*a*^Four factors include extraversion, neuroticism, emotional exhaustion, and voice behavior. ^*b*^We obtained the three-factor model by combining the items measuring extraversion and neuroticism to form a Time 1 factor. ^*c*^All items reported by employees were combined to form an employee–source factor, whereas voice behavior remained as another source factor. ^*d*^All measuring items were combined into one factor.

### Hypotheses Testing

We presented means, standard deviations, and correlations of all variables in Table 2.

**TABLE 2:** Means, standard deviations (SD), reliability coefficients, and correlations.

	Mean	SD	1	2	3	4	5	6	7	8
Gender^a^	0.66	0.47	−							
Age (years)	28.99	4.53	–0.03	−						
Education level^b^	1.91	0.49	−0.10*	–0.09	−					
Tenure (years)	6.79	4.88	0.04	0.90**	−0.27**	−				
Extraversion	4.52	0.80	−0.16**	0.01	0.05	0.01	(0.72)			
Neuroticism	2.90	0.92	0.08	–0.08	–0.04	–0.07	−0.23**	(0.79)		
Emotional exhaustion	3.41	1.38	0.12*	0.05	−0.10*	0.07	−0.26**	0.30**	(0.95)	
Employee voice	5.19	1.05	0.02	0.17**	0.05	0.17**	0.14**	0.01	−0.12*	(0.94)

*N* = 435. *p < 0.05. **p < 0.01 (two-tailed). Reliability coefficients for the scales in parenthesis along the diagonal. ^*a*^ Dummy coded: 0 = male, 1 = female. ^*b*^ Dummy coded: 1 = high school/college degree, 2 = undergraduate degree, 3 = graduate degree.

**FIGURE 1:**
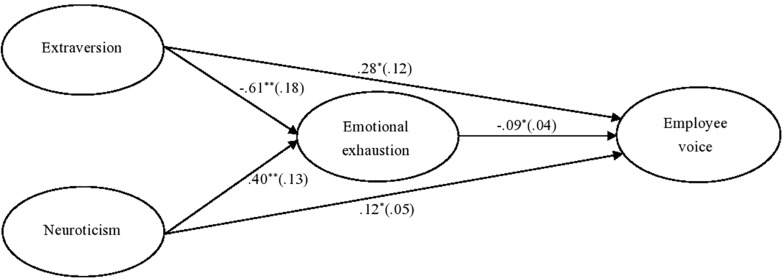
Multilevel structural equation modeling results for the hypothesized model. *N* = 435. *p < 0.05. **p < 0.01 (two-tailed). Unstandardized estimates of the path coefficients, with standard errors in parentheses.

By controlling for age, gender, education level, and organizational tenure, we first estimated a full mediation model in which there were no direct relationships between the independent variables and voice behavior. The full mediation model had good fit indices [χ^2^(229) = 484.85, *p* < 0.01, RMSEA = 0.05, CFI = 0.95, TLI = 0.94, SRMR = 0.05]. We then estimated a partial mediation model that added the above two direct effects. The results showed the partial mediation model achieved a similar model fit [χ^2^(227) = 477.38, *p* < 0.01, RMSEA = 0.05, CFI = 0.95, TLI = 0.94, SRMR = 0.05]. χ^2^ difference test between these two models (Δχ^2^(2) = 7.46, *p* < 0.05) indicated that adding the two direct paths from extraversion and neuroticism to employee voice significantly improves the overall model fit. In summary, our findings showed that emotional exhaustion partial mediated the relationships between the two personality variables and voice behavior. Figure 1 depicts significant paths and their unstandardized coefficient estimates for the hypothesized model.

Figure 1 shows that extraversion related negatively to emotional exhaustion (*b* = -0.61, SE = 0.18, *p* < 0.01), whereas neuroticism related positively to emotional exhaustion (*b* = 0.40, SE = 0.13, *p* < 0.01). Emotional exhaustion related negatively to employee voice (*b* = -0.09, SE = 0.04, *p* < 0.05). The direct effect from extraversion to employee voice was 0.28 (SE = 0.12, *p* < 0.05), whereas the direct effect from neuroticism to employee voice was 0.12 (SE = 0.05, *p* < 0.05). Furthermore, Monte Carlo simulation with 20,000 replications showed that the indirect effect from extraversion to voice behavior *via* emotional exhaustion was 0.06, with a 95% CI of [0.003, 0.137], and indirect effect from neuroticism to voice behavior *via* emotional exhaustion was -0.04, with a 95% CI of [-0.104, -0.002]. Because the CIs did not contain zero, the two indirect relationships were significant. Thus, H1 and H2 were supported. We further compared these two indirect effects. The result showed that these two indirect effects have significant difference (0.10, 95% CI [0.013, 0.196]).^1^ Additionally, the total effect from extraversion to employee voice was 0.34 (SE = 0.12, *p* < 0.01), whereas the total effect from neuroticism to employee voice was 0.08 (SE = 0.05, *p* < 0.1).

## Discussion

This study examined how extraversion and neuroticism, respectively, affect employee voice through the indirect effect of emotional exhaustion. Our analyses indicated that extraversion had a positive indirect effect on employee voice *via* emotional exhaustion, whereas neuroticism had a negative indirect effect on employee voice *via* emotional exhaustion.

### Theoretical Implications

Our study extends prior research on why some employees voice at work. Drawing from early evidence (e.g., LePine and Van Dyne, 2001; Nikolaou et al., 2008), we examined the indirect relationship between extraversion and neuroticism, respectively, and employee voice. Our findings indicate that extraversion and neuroticism could be antecedents of employee voice (Klass et al., 2012; Morrison, 2014). Our study reflects the complicated mechanism behind the relationship between Big-Five personality traits and employee voice (Klass et al., 2012). Our findings suggest how the Big-Five personality traits influence employee voice through the employees’ innate psychological process. We identified that, as a traditionally insignificant predictor, neuroticism could be activated to influence employee voice. By demonstrating the mediating role of emotional exhaustion, we agree that emotional stimuli may substantially explain variance of voice among employees (Morrison, 2014; Chamberlin et al., 2017). Our finding highlights the role of emotional stimuli in constructing the psychological process of employee voice (Morrison, 2011, 2014). In addition to fear, futility, image or career risks, certain new inhibitors relating to negative emotional stimuli deserve deep investigation (Morrison, 2014; Qin et al., 2014).

In particular, we identified a “suppressing” mediating role of emotional exhaustion through which both the absolute value and significance of the total effect of neuroticism on employee voice were reduced because of the reversed direction between the direct and indirect effect (MacKinnon et al., 2000; Shrout and Bolger, 2002; MacKinnon, 2008). Empirically, this finding provides additional evidence about certain neglected mediating mechanisms that may affect relationship between neuroticism and employee voice as either negative (LePine and Van Dyne, 2001; Nikolaou et al., 2008) or insignificant (Crant et al., 2011; Zare and Flinchbaugh, 2019). Theoretically, this finding implicitly shows another perspective on the personality–employee voice relationship. We used the COR theory to see voice as a sort of planned behavior in which individuals will voice after they evaluate that the benefits of voice exceed its cost (Morrison, 2011, 2014; Liang et al., 2012). As such, neurotic employees are more likely to be emotionally exhausted and accordingly are reluctant to voice for reserving personal resources. Instead, voice scholars have recently introduced a view of non-conscious process (Morrison, 2014; Lam et al., 2018) through which neurotic employees may express directly when they have any constructive ideas because they are difficult to control for and adjust their emotions. In summary, our finding indicates a complicated relationship between personality traits and employee voice and accordingly encourages more deep investigation.

Our study also contributes to the COR theory. We followed the most significant trend of the COR theory relating to “better understand how individuals allocate and conserve resources in the context of resource gains and loss” (Hobfoll et al., 2018). We took employee voice as a means of resource investment strategies that may conserve personal resources to reduce resource losses derived from emotional exhaustion. Essentially, while resources in COR could be broadly defined as “anything perceived by the individual to help attain his or her goals” (Halbesleben et al., 2014, p. 1338), our findings note the importance of clarifying the nature of personal resources (Halbesleben et al., 2014; Hobfoll et al., 2018). By examining the indirect role of emotional exhaustion, we illustrate that emotional resources are key to persons (Halbesleben et al., 2014; Hobfoll et al., 2018). The significantly opposite emotional effects of the two personality traits in our study echo LePine and Van Dyne’s (2001) advice that emotional stimuli have different impacts in employee voice and explain that the various effects of the two Big-Five personality traits on employee voice result from negative emotional mechanisms. This finding encourages future research to specify how employee voice may vary across multiple natures of dispositional resources in terms of negative emotional mechanisms.

Additionally, our work suggests that emotional resource losses may implicitly help understand the degree of emotional exhaustion, and meanwhile, such an emotional strain ultimately motivates employee voice to avoid further losses of their emotional resources. As such, emotional resources are low in the stressful conditions that employee voice is seen as a coping behavior of resource consumption. This finding shows that the value of personal resources can vary significantly in terms of emotional resources individuals perceive and the psychological process by which individuals conserve and/or acquire these resources (Halbesleben et al., 2014). Future studies may clarify the nature of personal resources and specify their underlying mechanism before evaluating their values to employee voice.

### Managerial Implications

Our findings provide certain guidance for managerial practitioners. We found that extraversion and neuroticism are important factors to influence employee voice. This finding somewhat indicates that all the employees are possible to speak up constructively in the workplace. Thus, organizations should focus on increasing the overall employee qualities rather than simply relying on some employees to speak up constructively. An open organizational culture could cultivate a high level of senses of belongings through which employees with differently individual characteristics would like to voice for enhancing organizational effectiveness. In addition, we recommend encouraging more emotional regulation practices in organizations as a means for helping employees manage emotions. A more open culture could improve direct communication channels, which in turn may facilitate healthier organizational operation and make efficient resource allocation to stimulate constructive advice from employees.

Second, organizations and managers should manage employees wisely. Organizational selection and recruitment process could introduce some psychological tests or face-to-face interviews to categorize each job candidate. Newcomer orientation programs provide another excellent opportunity to recognize each employee at an early stage. Extravert employees may stand out in teambuilding games, while neurotic employees may be those who show concern and nervous in team collaboration presentations. Then, job designs could be assigned more appropriately in terms of the fit between employees’ personality characteristics and their potentials contributions for voice. For example, extravert and emotional stable employees would like to share their thoughts and thus may stand out with work such as marketing and helpdesks that need much opening communication and team participation. In contrast, introvert and neurotic employees are inclined to work at back offices and accounting that follow more regular routines. Furthermore, voice development and motivation policies are highly recommended in terms of the employees’ personal characteristics. For example, extravert employees could be paired with coaches who provide professional feedback and pragmatic rewards in appreciation for their constructive feedback. Instead, neurotic employees require supportive, patient, and emotional attached mentors who sincerely recognize the contributions of these neurotic employees’ advice.

Third, every employee could express constructive comments through self-regulation and honest communication. Employees can follow their own approaches to voice. While extravert employees actively voice their concerns when emotionally exhausted, these reporters should learn to think twice and present their advice based on reasonable claims and evidence. By contrast, neurotic employees should not wait until they feel emotionally depleted. They could confess their vulnerability with human resource managers or direct leaders and seek to constructive solution by following organizational regulations.

### Implications for the General Public

Policy makers could build institutional environments for social welfare to establish a well-functioning public sector for civil servants. Policy makers could revise relevant tax regulations to reduce the drivers of public sectors in pursuit of much effectiveness and efficiency. In doing so, the overall quality of civil servants’ lives will increase. Policy makers could also enact legal protection to authorize civil servants to voice at work. Collective voice strategies could be introduced in the settings of collectivistic cultures such as China where individuals will decrease their voice if they are emotionally exhausted. Furthermore, policy makers could improve employment relationship in two aspects. Promoting economic development and labor markets would provide more job opportunities for public services that help civil servants choose certain coping strategies such as voice and exit if they are suffering at work. Regulating labor contracts can set boundaries of work responsibilities that reasonably protect the well-being of civil servants. While public organizations could not endlessly request prosocial voice for the interests of public organizations and communities, civil servants could appropriately enhance their discretionary contribution to voice.

Our finding also offers three suggestions on how public organizations manage their voice systems to inspire civil servants’ commitment to the public interest. First, organization configuration could be designed wisely. Our study indicates that a diversity of individuals could voice. Thus, public sectors could be more organic and flexible to benefit for a larger groups of civil servants. Second, governance structure should be set carefully. Governance structure determines the extent to which interests of community, public sectors, and civil servants would be included in public organizations’ daily decision making. Also, governance structure influences the means of participation and the degree of empowerment. Both of them affect the scope and depth of prosocial voice in public organizations. Third, win–win voice strategies could be executed properly. Constructive feedback and timely rewards are useful to relive the concerns of civil servants if they suffer from burnout and thereby encourage them to reciprocate with prosocial voice.

Finally, our study advises how civil servants take the role of citizenship in public sectors. We implicate the potential dark side of prosocial voice by identifying the vulnerability of individuals who voice prosocially. We suggest the existence of potential interest conflicts among civil servants, public organizations, and social good. This is particularly significant for those (e.g., nurse, social workers) who are professionally occupational citizenship. As such, civil servants should also consider certain strategies to enhance their own quality of lives. Organizing a self-management union and other organizational structures may inspire these citizens in pursuing a balance of in-role and extra-role of prosocial voice. Maintaining an appropriate attitude is another way. Civil servants do not overestimate the prosocial aspect of voice. Rather, they should remind themselves that prosocial voice is discretionary and common in the daily work life.

### Limitations and Future Direction

This study has certain limitations that request future research to address. First, we could not completely confirm the causality of our findings. We recommend future studies to conduct laboratory and experiment designs. Second, our research context in a Chinese state-owned bank led to an issue about the generalizability of our findings. We expect more evidence to replicate our model in other organizational, industrial, and cultural settings. Third, the estimates of our model fits were acceptable. We found that the reason was partially due to reversed items we adopted to measure the two Big-Five personality traits. Although we used the parcel technique to confirm the reliability and construct validity of our findings, future research may use alternative measurement tools.

Additionally, researchers could better investigate the relationship between Big-Five personality traits and employee voice. Employee voice could be categorized in terms of its content, and accordingly have a complex relationship with Big-Five personality traits. Future work could examine the psychological mechanisms that connect Big-Five personality traits with other forms of employee voice. Furthermore, researchers may examine boundary conditions that moderate the individual disposition–employee voice relationship. The COR theory has prioritized these contextual factors as external resources that may interact with internal resources to influence work behaviors (Halbesleben et al., 2014; Hobfoll et al., 2018). Therefore, employees with the same degree of Big-Five personality traits may exhibit different magnitudes in employee voice across tasks, work relationships, and organizational settings. We expect more studies to specify boundary situations that influence voice of employees who exhibit unique personality traits.

## Conclusion

Research has been interested in “who does (not)speak up at work?” over the past two decades. But the relationship between Big-Five personality traits and employee voice is in needs of better understanding given a lack of exploration on a “why” question about both underlying mediating mechanisms and fresh theoretical perspectives. By introducing the COR theory, we examined and found the indirect effect of emotional exhaustion between the two Big-Five personality traits and employee voice. Although more efforts are needed to enhance the causality and generalizability of our findings, we expect that our study could provide some thoughts and inspire a better understanding of the innate psychological mechanism through which individual dispositions affect employee voice.

## Data Availability Statement

The datasets generated for this study are available on request to the corresponding author.

## Ethics Statement

All procedures performed in studies involving human participants were in accordance with the ethical standards of the institutional and/or national research committee and with the 1964 Helsinki declaration and its later amendments or comparable ethical standards. This article does not contain any studies with animals performed by any of the authors. Informed consent was obtained from all individual participants included in the study.

## Author Contributions

JL and SX together designed the study and wrote the manuscript. JL collected the data, and completed the statistical analysis. Both authors contributed to the article and approved the submitted version.

## Conflict of Interest

The authors declare that the research was conducted in the absence of any commercial or financial relationships that could be construed as a potential conflict of interest.

## Footnotes

1 We appreciated an reviewer for giving us this comment and clear guidance.
